# Association of Wealth With Longevity in US Adults at Midlife

**DOI:** 10.1001/jamahealthforum.2021.1652

**Published:** 2021-07-23

**Authors:** Eric D. Finegood, Daniel A. Briley, Nicholas A. Turiano, Alexa Freedman, Susan C. South, Robert F. Krueger, Edith Chen, Daniel K. Mroczek, Gregory E. Miller

**Affiliations:** 1 Institute for Policy Research, Northwestern University, Evanston, Illinois; 2 Department of Psychology, Northwestern University, Evanston, Illinois; 3 Department of Psychology, University of Illinois Urbana-Champaign, Urbana; 4 Department of Psychology, West Virginia University, Morgantown; 5 Department of Psychological Sciences, Purdue University, West Lafayette, Indiana; 6 Department of Psychology, University of Minnesota, Minneapolis; 7 Department of Medical Social Sciences, Northwestern University, Evanston, Illinois

## Abstract

**Importance:**

Wealthy adults tend to live longer than those with less wealth. However, a challenge in this area of research has been the reduction of potential confounding by factors associated with the early environment and heritable traits, which could simultaneously affect socioeconomic circumstances in adulthood and health across the life course.

**Objective:**

To identify the association between net worth at midlife and subsequent all-cause mortality in individuals as well as within siblings and twin pairs.

**Design, Setting, and Participants:**

This cohort study conducted a series of analyses using data from the Midlife in the United States (MIDUS) study, an ongoing national study of health and aging. The sample included adults (unrelated individuals, full siblings, and dizygotic and monozygotic twins) aged 20 to 75 years, who participated in wave 1 of the MIDUS study, which occurred from 1994 to 1996. The analyses were conducted between November 16, 2019, and May 18, 2021.

**Exposures:**

Self-reported net worth (total financial assets minus liabilities) at midlife (the middle years of life).

**Main Outcomes and Measures:**

All-cause mortality was tracked over nearly 24 years of follow-up, with a censor date of October 31, 2018. Survival models tested the association between net worth and all-cause mortality. Discordant sibling and twin analyses compared longevity within siblings and twin pairs who, given their shared early experiences and genetic backgrounds, were matched on these factors.

**Results:**

The full sample comprised 5414 participants, who had a mean (SD) age of 46.7 (12.7) years and included 2766 women (51.1%). Higher net worth was associated with lower mortality risk (hazard ratio [HR], 0.95; 95% CI, 0.94-0.97; *P* < .001). Among siblings and twin pairs specifically (n = 2490), a similar within-family association was observed between higher net worth and lower mortality (HR, 0.94; 95% CI, 0.91-0.97; *P* = .001), suggesting that the sibling or twin with more wealth tended to live longer than their co-sibling or co-twin with less wealth. When separate estimates were performed for the subsamples of siblings (HR, 0.94; 95% CI, 0.90-0.97; *P* = .002), dizygotic twins (HR, 0.94; 95% CI, 0.86-1.02; *P* = .19), and monozygotic twins (HR, 0.95; 95% CI, 0.87-1.04; *P* = .34), the within-family estimates of the net worth–mortality association were similar, although the precision of estimates was reduced among twins.

**Conclusions and Relevance:**

This cohort study found that wealth accumulation at midlife was associated with longevity in US adults. Discordant sibling analyses suggested that this association is unlikely to be simply an artifact of early experiences or heritable characteristics shared by families.

## Key Points

**Question:**

Is net worth at midlife associated with all-cause mortality?

**Findings:**

In this cohort study of 5414 participants in the Midlife in the United States study, those who had accumulated a higher net worth by midlife had significantly lower mortality risk over the subsequent 24 years. In sibling and twin comparison models that controlled for shared early life experiences and genetic influence, the association between net worth and longevity was similar in magnitude.

**Meaning:**

Net worth at midlife was associated with longevity among adults in the study, and this association is unlikely to be merely an artifact of early experiences or heritable traits shared by families.

## Teaser

This cohort study examines the shared environmental and genetic characteristics as well as the mortality risk of adult individuals, siblings, and twins participating in a longitudinal study.

## Introduction

Socioeconomic disparities in life expectancy are substantial in size.^1,2,3^ Financial wealth or net worth, which is the value of an individual’s assets (such as savings, real estate, and vehicles) minus liabilities,^4,5^ is directly associated with longevity^4,6,7,8,9^ and, in some studies, has been found to be more strongly associated with mortality than other indicators of socioeconomic status, such as occupational prestige, educational attainment,^7^ and income.^6^ However, a challenge in this area of research has been eliminating or minimizing the potential for confounding by the early environment and heritable traits, either of which could simultaneously affect socioeconomic conditions in adulthood and health in the course of life.^10^

Discordant sibling designs allow for the identification and control of such confounders. Full siblings who were raised in the same family share much of their early rearing environment and are genetically related to one another. Thus, in sibling-comparison studies, factors that are shared between siblings are controlled.^11,12^ Twin comparisons provide an even greater control of family-level early-life confounding and, in the case of monozygotic (MZ) twins, control for all heritable genetic factors.^13,14^ Previous research found that discordance in occupational prestige was associated with cardiovascular risk^15^ and overall mortality^16^; twins with lower-prestige jobs had worse health on both outcomes compared with their co-twins with higher-prestige jobs. This pattern suggests that socioeconomic disparities in health are affected by experiential factors in adulthood over and above any potential confounders that involve the siblings’ shared early environment and genetic characteristics. In other discordant sibling and twin analyses, educational attainment^16,17,18,19,20,21,22^ and composite measures of adult socioeconomic position^23^ also have been associated with better adult health outcomes^18,22,23^ and longevity.^16,17,19,20,21^ However, results from these and other studies that used different methods do suggest these associations may be partially explained by shared family-level environmental factors^17,18,19,21,22^ or genetic predispositions.^10,18,23^

Comparatively little attention has been given to wealth disparities, a potentially important oversight because wealth inequality is far greater and growing at a faster rate than income inequality in the United States.^24,25^ At the individual level, those with greater wealth are better able to access health-promoting resources (eg, medical care, safe places to exercise, and fresh foods). Wealthy individuals also have more protection from economic shocks, such as job loss, unexpected health care expenses, or other financial crises. The association between wealth and longevity has become more relevant in the past year because of the economic fallout from the COVID-19 pandemic, which has disproportionately affected the financial security of low-income^26^ and older workers.^27^

In this cohort study, we used a discordant sibling design to conservatively estimate the association between wealth and longevity. Specifically, we aimed to identify the association between net worth at midlife (the middle years of life) and subsequent all-cause mortality in individuals as well as within siblings and twin pairs. We posed 2 research questions. First, was wealth accumulation at midlife associated with longevity over a nearly 24-year follow-up? Consistent with previous work,^4,6,7,8,9,28^ we expected that higher wealth accumulation would be associated with increased longevity. Second, was the wealth-longevity association present over and above controls for family and heritable factors that could confound this association? That is, was there evidence of a within-family association between wealth and longevity among siblings and twins in the same family? Alternatively, was the wealth-longevity association primarily driven by common factors at the family level, presumably involving early experiences and/or heritable factors?

## Methods

The analyses in this cohort study were conducted between November 16, 2019, and May 18, 2021, and were preregistered on the Open Science Framework^29^ on November 15, 2019. The preregistration included hypotheses, decision rules for inclusion, rationale for covariates, and statistical plan. Deviations from the original analysis plan are detailed in eMethods 3 in the Supplement. We followed the Strengthening the Reporting of Observational Studies in Epidemiology (STROBE) reporting guideline.^30^ The institutional review boards of the University of Wisconsin-Madison and Harvard Medical School approved the Midlife in the United States (MIDUS) study procedures. Participants in the MIDUS study provided oral informed consent. The institutional review board approval and informed consent for the MIDUS study extend to the present study, which used publicly available MIDUS data, including all mortality data up to 2016.

### Participants and Analysis Sample

The data used were obtained from the MIDUS^31^ study, an ongoing national study of health and aging that began in 1994. In the present study, we used data from wave 1 of the MIDUS study (MIDUS 1), which were collected in 1994 to 1996 from 7108 adults aged 20 to 75 years. These participants were recruited through a nationally representative random-digit dialing sampling strategy that included subsamples of siblings and twins. Participants who provided oral informed consent completed a telephone-assisted survey and a mailed self-administered questionnaire.

Mortality data through October 31, 2018, were collected by the MIDUS study team at the University of Wisconsin-Madison. Of the 7108 cases from MIDUS 1, 7017 were considered for inclusion in the current analysis. We selected cases with complete data on mortality status, net worth, and relevant covariates (n = 5414). These cases included 2675 individuals (designated here as singletons) who did not have a sibling or co-twin in the MIDUS study, 1282 nontwin full siblings, 864 dizygotic (DZ) twins, and 593 MZ twins. Twins or siblings who had complete data and who were in a pair with a co-sibling or co-twin who were missing data were included in the full analytic sample. However, they were excluded from subsequent discordant twin or sibling analyses, resulting in 1214 nontwin full siblings, 740 DZ twins, and 536 MZ twins in the discordant twin or sibling analyses. The eMethods 1 in the Supplement provides details on the eligibility criteria and missing data.

### Measures

At MIDUS 1, participants responded to the following question: “Suppose you (and your spouse or partner) cashed in all your checking and savings accounts, stocks and bonds, real estate, sold your home, your vehicles, and all your valuable possessions. Then suppose you put that money toward paying off your mortgage and all your other loans, debts, and credit cards. Would you have any money left over after paying your debts or would you still owe money?” Participants reported how much that amount would be, using binned response categories that specified ranges of dollar amounts. Amount of money owed was truncated at a value of $0. Net worth that exceeded $1 million was truncated at that value.

Covariates included parental educational level and participant age, self-reported race/ethnicity (analyzed here as non-White vs White), sex (female vs male), history of cancer or heart disease as diagnosed by a medical doctor, and status of ever smoking cigarettes regularly or consuming alcohol regularly (the eMethods 1 in the Supplement provides coding details). At MIDUS 1, participants self-reported on their race. Response options included White, Black and/or African American, Native American or Aleutian Islander, Asian or Pacific Islander, multiracial, or other race. Because of the low number of individuals who identified as a race other than White, we dichotomized race categories into non-White vs White. In addition, data on self-identification as Hispanic or Latino were not available at MIDUS 1.

Mortality follow-up was completed by the MIDUS study team at the University of Wisconsin-Madison. Date of death was obtained from various sources, including relative responses, other informant reports, newspaper or online obituaries, and the National Death Index (using the 15th day of the month, rather than the exact day of death, to maintain participant confidentiality). Survival time was the number of years between the date when the MIDUS 1 self-administered questionnaires were returned to the MIDUS study team (1994 to 1996) and the date of death; if the participant was alive, the censor date was October 31, 2018, which was the date of the MIDUS study team’s latest mortality update.

### Statistical Analysis 

A series of Cox proportional hazards regression models for survival analyses were estimated using Stata, version 16 (StataCorp LLC).^32^ In model 1, we tested the individual-level association between wealth and longevity by conducting survival analysis among all cases who had complete data on the analysis variables and by using robust SEs to account for dependence among family members.

Next, we estimated survival models only within the subsample of twins and nontwin siblings. In model 2, siblings and twins were treated as individuals, but a shared frailty was included to estimate dependence among family members. In model 3, we estimated the within-family association between wealth and longevity by calculating the family-level mean net worth (among families that had ≥2 members with complete data) and subsequently calculating the difference between each individual’s net worth and their family’s mean net worth. This between-within method is a common approach to fixed-effects modeling.^33,34^ When applied in this way, the between-within method allowed us to compare siblings or twins in the same family to one another and thus to control for all unmeasured shared family-level variables consistent with the discordant sibling design.^13,14,35^ In survival analysis, the between-within method has been shown to provide similar estimates to more common approaches for co-sibling or co-twin control (eg, conditional likelihood methods like stratified Cox regression) and has been observed to be optimal statistically.^34^

To further disambiguate environmental vs genetic influences, we tested whether within-family associations between net worth and longevity varied across the nontwin sibling, MZ twin, and DZ twin subsamples. We included a pair of 2-way interaction terms crossing the net worth mean deviation scores with dummy codes for DZ twins or MZ twins. A Wald test was used to measure the equality of the within-family net worth coefficient across siblings, DZ twins, and MZ twins. We estimated separate survival models for nontwin siblings, DZ twins, and MZ twins (models 4-6). A significant within-family association (2-sided *P* < .05) that was observed among nontwin siblings but not among DZ or MZ twin pairs would suggest residual confounding by early life factors because twins share a closer prenatal and postnatal environment than nontwin siblings. A within-family association that was observed among both siblings and DZ twin pairs but not among MZ twin pairs would suggest genetic confounding. Sensitivity analyses addressed the skewed distribution of net worth and tested the possibility of nonlinear associations between net worth and longevity. Sensitivity analyses also clarified the role of preexisting health problems and considered other model specifications.

Primary models were reestimated as stratified Cox regressions using Stata^32^ (eMethods 2 in the Supplement) and as multilevel Cox regressions using Mplus (Muthén & Muthén) (eTables 3-5 in the Supplement).^36^ A 2-sided *P* < .05 was considered statistically significant.

## Results

A total of 5414 participants from MIDUS 1 were included in the full analysis sample. These participants had a mean (SD) age of 46.7 (12.7) years; 2766 were women (51.1%), 2648 were men (48.9%), and 4927 (91.0%) self-identified as White individuals. Participants had a mean (SD) net worth of $122 153 ($209 537; median, $32 500). Of these participants, 675 (12.5%) had been diagnosed with a heart problem and 381 (7%) had a cancer diagnosis. In addition, 2790 participants (51.5%) reported ever having smoked cigarettes regularly and 2301 participants (42.5%) reported having used alcohol regularly. Table 1 displays the descriptive statistics, by subsamples of singletons, nontwin siblings, DZ twins, and MZ twins. By the censor date (October 31, 2018), 1010 individuals (18.7%) in the full sample had died. Fifty percent of siblings and twins were in families whose members differed by less than or equal to $87 500 in net worth at MIDUS 1 (interquartile range=$212 500).

**Table 1.** Descriptive Statistics of the Analysis Variables

Variable	No. (%)				
	Full sample (n = 5414)	Singleton (n = 2675)	Nontwin full sibling (n = 1214)	DZ twins (n = 740)	MZ twins (n = 536)
Net worth, mean (SD), $	122 153.02 (209 537.49)	111 425.23 (202 341.67)	160 577.84 (240 643.26)	97 760.81 (167 631.37)	131 624.06 (217 415.75)
Age, mean (SD), y	46.7 (12.7)	46.2 (13.0)	49.3 (12.4)	45.9 (12.1)	44.7 (11.7)
Sex					
Female	2766 (51.1)	1267 (47.4)	667 (54.9)	409 (55.3)	282 (52.6)
Male	2648 (48.9)	1408 (52.6)	547 (45.1)	331 (44.7)	254 (47.4)
Race					
White	4927 (91)	2334 (87.3)	1161 (95.6)	706 (95.4)	503 (93.8)
Black or African American	255 (4.7)	173 (6.5)	23 (1.9)	23 (3.1)	20 (3.7)
Other^a^	232 (4.3)	168 (6.3)	30 (2.5)	11 (1.5)	13 (2.4)
Heart disease	675 (12.5)	315 (11.8)	172 (14.2)	104 (14.1)	58 (10.8)
Cancer	381 (7.0)	165 (6.2)	126 (10.4)	43 (5.8)	26 (4.9)
Regular cigarette smoking	2790 (51.5)	1464 (54.7)	587 (48.4)	362 (48.9)	242 (45.1)
Regular alcohol use	2301 (42.5)	1204 (45.0)	508 (41.8)	301 (40.7)	186 (34.7)
Parental educational level					
<High school diploma	1275 (23.6)	744 (27.8)	182 (15.0)	184 (24.9)	106 (19.8)
High school diploma	1937 (35.8)	983 (36.7)	433 (35.7)	252 (34.1)	176 (32.8)
Some college, 2-y associate’s degree, or vocational school	873 (16.1)	368 (13.8)	229 (18.9)	110 (14.9)	118 (22.0)
4-y Bachelor’s degree or some graduate school	822 (15.2)	371 (13.9)	215 (17.7)	126 (17.0)	80 (14.9)
Master’s or professional degree	507 (9.4)	209 (7.8)	155 (12.8)	68 (9.2)	56 (10.4)

Abbreviations: DZ, dizygotic; MZ, monozygotic.

^a^ Other race included multiracial, Native American or Aleutian Islander, Asian or Pacific Islander, and Other.

### Cohort Analyses

Survival models were run as gamma shared frailty Cox regressions, stratified Cox regressions, and multilevel Cox regressions. Regardless of the model used, the estimates were similar (eMethods 2 and 3 in the Supplement). Thus, results from the shared frailty Cox regressions are described herein.

Results from the full sample (model 1; n = 5414; 1010 deaths) are presented in Table 2. After covariate adjustment, net worth was inversely associated with mortality (HR, 0.95; 95% CI, 0.94-0.97; *P* < .001) such that the hazard was 5% lower for every additional $50 000 of net worth accumulated at midlife. In the subsample of siblings and twins (model 2; n = 2490; 421 deaths), the net worth HR was similar (HR, 0.95; 95% CI, 0.93-0.97; *P* < .001).

**Table 2.** Cox Proportional Hazards Regression for Survival Models

Model^a^	HR (95% CI)^b^	*P* value
**Model 1: full sample (n = 5414)**		
Age	1.10 (1.09-1.11)	<.001
Female sex	0.82 (0.72-0.94)	.005
Non-White race^c^	1.13 (0.87-1.46)	.35
Parental education	0.97 (0.95-0.99)	.03
Heart disease	1.87 (1.61-2.17)	<.001
Cancer	1.44 (1.22-1.70)	<.001
Cigarette smoking	1.75 (1.53-2.01)	<.001
Alcohol use	1.04 (0.91-1.19)	.47
Net worth	0.95 (0.94-0.97)	<.001
**Model 2: siblings and twins (n = 2490)**		
Age	1.10 (1.09-1.12)	<.001
Female sex	0.76 (0.61-0.95)	.01
Non-White race^c^	0.85 (0.48-1.52)	.60
Parental education	0.97 (0.93-1.01)	.17
Heart disease	1.93 (1.54-2.43)	<.001
Cancer	1.55 (1.17-2.06)	.002
Cigarette smoking	2.08 (1.66-2.61)	<.001
Alcohol use	0.92 (0.73-1.15)	.47
Net worth	0.95 (0.93-0.97)	<.001
**Model 3: siblings and twins (n = 2490)**		
Age	1.10 (1.09-1.11)	<.001
Female sex	0.76 (0.61-0.95)	.01
Non-White race^c^	0.87 (0.49-1.54)	.64
Parental education	0.97 (0.93-1.01)	.15
Heart disease	1.94 (1.54-2.43)	<.001
Cancer	1.56 (1.18-2.07)	.002
Cigarette smoking	2.09 (1.66-2.62)	<.001
Alcohol use	0.91 (0.73-1.14)	.43
Net worth		
Between family	0.96 (0.93-0.99)	.01
Within family	0.94 (0.91-0.97)	.001
**Model 4: nontwin siblings (n = 1214)**		
Age	1.10 (1.08-1.12)	<.001
Female sex	0.64 (0.47-0.86)	.004
Non-White race^c^	1.51 (0.72-3.14)	.27
Parental education	1.00 (0.94-1.06)	.88
Heart disease	2.09 (1.52-2.86)	<.001
Cancer	1.92 (1.35-2.72)	<.001
Cigarette smoking	2.21 (1.61-3.04)	<.001
Alcohol use	0.73 (0.53-1.00)	.05
Net worth		
Between family	0.98 (0.94-1.02)	.38
Within family	0.94 (0.90-0.97)	.002
**Model 5: DZ twins (n = 740)**		
Age	1.12 (1.09-1.14)	<.001
Female sex	0.88 (0.59-1.31)	.53
Non-White race^c^	0.25 (0.06-1.02)	.05
Parental education	0.96 (0.90-1.03)	.37
Heart disease	1.72 (1.14-2.57)	.009
Cancer	1.01 (0.57-1.79)	.96
Cigarette smoking	2.08 (1.38-3.14)	<.001
Alcohol use	1.18 (0.80-1.75)	.39
Net worth		
Between family	0.90 (0.84-0.97)	.009
Within family	0.94 (0.86-1.02)	.19
**Model 6: MZ twins (n = 536)**		
Age	1.11 (1.08-1.14)	<.001
Female sex	1.02 (0.60-1.73)	.93
Non-White race^c^	1.17 (0.33-4.14)	.79
Parental education	0.91 (0.83-1.01)	.10
Heart disease	2.37 (1.27-4.42)	.006
Cancer	1.53 (0.61-3.81)	.35
Cigarette smoking	2.05 (1.19-3.54)	.009
Alcohol use	1.01 (0.59-1.71)	.97
Net worth		
Between family	0.96 (0.89-1.02)	.25
Within family	0.95 (0.87-1.04)	.34

Abbreviations: DZ, dizygotic; HR, hazard ratio; MZ, monozygotic.

^a^ The models are described in the Statistical Analysis subsection of the Methods section.

^b^ The HR for net worth of both between-family and within-family associations reflects a difference of $50 000.

^c^ Non-White race included Black and/or African American, Native American or Aleutian Islander, Asian or Pacific Islander, multiracial, and other.

### Between-Within Models

**Figure 1.**
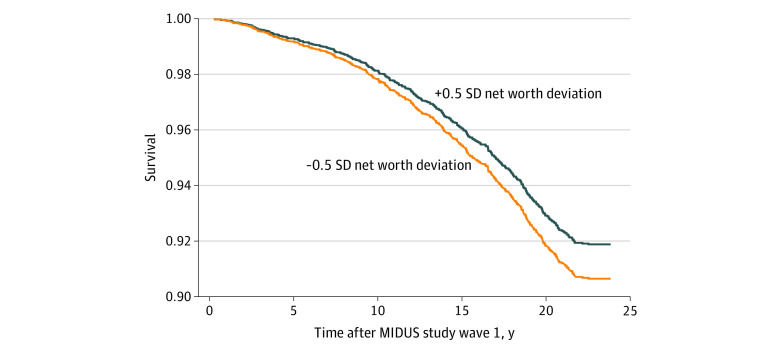
Within-Family Association Between Net Worth and Longevity The survival curves represent 2 family members whose net worth differed by approximately $139 000 at Midlife in the United States (MIDUS) study wave 1; this amount corresponded to within-family net worth deviation scores that are ±0.5 SD from the mean deviation score of 0.00. The survival curves were adjusted for family-level mean net worth as well as participant age at wave 1 of the MIDUS study, race/ethnicity, sex, history of cancer or heart disease, health behaviors, and parental educational level.

Similar to models 1 and 2, the between-within model among siblings and twins (model 3) suggested that net worth was inversely associated with mortality within families (HR, 0.94; 95% CI, 0.91-0.97; *P* = .001). These patterns suggested that a sibling or twin who had accumulated more net worth by midlife tended to live longer than their co-siblings or co-twin with less net worth. Figure 1 depicts survival curves that were split at values above and below 0.5 of an SD from the mean deviation net worth score (mean of 0.00). In Figure 1, the net worth–mortality association is conditional on the shared frailty, thus the lines represent the estimated survival of 2 family members whose net worth differed by 0.5 SD or $139 000 and who were approximately 47 years of age at MIDUS 1 (with all other variables held at their means). A difference of $139 000 in net worth was associated with a 13% relative decrease in the probability of death nearly 24 years later, favoring the family member with a higher net worth; given the low base rate of mortality in the sample, this decrease translated into more than a 1% absolute difference in survival. The interactions between sibling type (ie, nontwin siblings, DZ twins, and MZ twins) and net worth deviation score were not significant, and the Wald test indicated that differences in the within-family HRs across sibling types were no greater than chance (χ^2^(2) = 0.05; *P* = .97).

**Figure 2.**
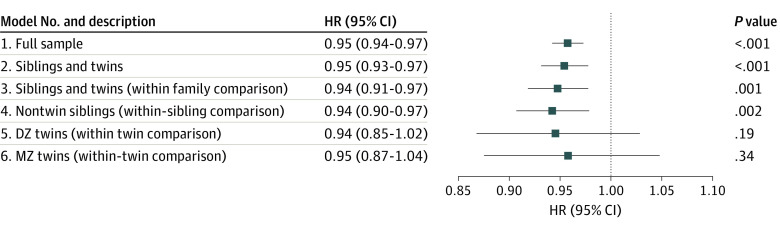
Hazard Ratios (HRs) and 95% CIs for the Net Worth Estimate Across Survival Models In each model, the HR reflects the decrease in hazard associated with a $50 000 increase in net worth. The squares represent the HR estimates, and the lines represent the 95% CIs. DZ indicates dizygotic; MZ, monozygotic.

Separate survival models were estimated for nontwin siblings (model 4), DZ twins (model 5), and MZ twins (model 6). Consistent with the Wald test, the within-family net worth estimates for each of the 3 subsamples were similar (nontwin siblings: HR, 0.94 [95% CI, 0.90-0.97; *P* = .002]; DZ twins: HR, 0.94 [95% CI, 0.86-1.02; *P* = .19]; MZ twins: HR, 0.95 [95% CI, 0.87-1.04; *P* = .34]). These HRs were also similar in magnitude to the net worth HR observed in the individual-level analyses (models 1 and 2). Among nontwin siblings, the *P* value for the within-family net worth estimate was significant at *P* < .05, although the *P* values were not significant in the DZ twin and MZ twin subsamples, likely because of the substantial decrease in the sample size and the number of deaths within the DZ twin (n = 118 decedents) and MZ twin (n = 79 decedents) subsamples. Figure 2 depicts the HRs and 95% CIs for the net worth estimate in the full sample (model 1) and sibling and twin subsample (model 2), the within-family comparison in the sibling and twin subsample (model 3), and the within-sibling and within-twin comparisons (models 4, 5, and 6).

### Sensitivity Analyses

The eMethods 2 and eTables 1-5 in the Supplement present the results of the sensitivity analyses conducted in the combined sibling and twin subsample. Briefly, these analyses found that the observed wealth-longevity association (1) was not an artifact of the right-skewed distribution of net worth, (2) had a roughly linear shape for most of the sample, and (3) was not explained by lifestyle practices and health conditions at MIDUS 1. For example, when net worth was recoded into deciles, the within-family association between net worth and mortality remained statistically significant (HR, 0.92; 95% CI, 0.87-0.96; *P* = .001). In addition, spline models (eTable 1 in the Supplement) suggested that the HR for the net worth–mortality association was similar for approximately 90% of participants in the full sample. Moreover, when restricting the full sample to only the siblings and twin pairs who were free of previous cancer or heart disease, the within-family association between net worth and mortality remained statistically significant (HR, 0.94; 95% CI, 0.90-0.98; *P* = .01). The eMethods 2 in the Supplement provides a complete description of all sensitivity analyses.

## Discussion

In contrast to most previous studies that examined associations between socioeconomic position and longevity using between-person analytic approaches, the present cohort study examined within-family associations between wealth and longevity— a more conservative test of the hypothesis because it implicitly controlled for all factors (eg, early experience and heritable characteristics) shared by siblings and twins. Findings from the within-family analyses suggested that wealth accumulation at midlife may be associated with longevity among adults in the United States. We believe that investigations such as the kind we conducted are important because of the near impossibility of performing an experimental study of the wealth-longevity association. Findings from this study also converged with the results from other studies that used different methods. For example, an analysis found that mortality risk among retired stockholders increased in the years after wealth shocks (ie, acute reductions in wealth) owing to exogenous stock market fluctuations.^28^

Across models, the associations observed between net worth and longevity were modest: we observed a 1% absolute difference in the probability of survival after nearly 24 years between family members who differed by approximately $139 000 in net worth at midlife. However, the MIDUS sample was relatively young (mean age of approximately 70 years) with relatively low mortality (18.7% of the full sample had died by the censor date). Thus, follow-up analyses are needed to ascertain whether the magnitude of these associations would change as mortality increases.

The associations between net worth and longevity in the DZ and MZ twin pairs were similar in magnitude to the associations observed in nontwin siblings. However, when the analyses were separated into subsamples of siblings, DZ twins, and MZ twins, the within-family net worth associations among DZ twins and MZ twins were not statistically significant. This finding was likely the result of lower power and reduced precision in the twin subsamples, as reflected in the wide CIs around the within-family estimates. However, it could also suggest the presence of confounding by shared environmental features. Other studies have discussed the difficulty in distinguishing null associations from false-negative results in sibling study designs when power varies widely between subsamples.^37^

The findings of this study suggest that the association between wealth at midlife and longevity is unlikely to be a simple artifact of environmental and heritable characteristics shared by siblings and twins. Moreover, the findings suggest that policies to support individuals’ ability to accrue wealth and to achieve financial security in adulthood could have considerable health benefits. These findings should be interpreted through a broader societal lens. The US ranks first in economic inequality among high-income nations.^38^ Over the past 30 years, the wealth gap between the high-income and low-income people in the US has widened through policies and practices that have diverted a substantial and increasing share of wealth from the lower- and middle-income groups to the affluent group.^24,38^ Such redistribution may have implications for longevity patterns in the coming decades. Policies to reduce the wealth gap, if implemented, could be expected to generate substantial returns to public health.

### Limitations

This study has several limitations. First, although the discordant sibling design, as we have modeled it, reduced confounding by shared environmental and heritable characteristics, it cannot elucidate whether wealth itself is a causal actor or a marker of other nonshared factors (eg, self-regulatory capacity and cognitive ability) and nonshared experiences (eg, social, educational, and professional trajectories) that covary with and/or contribute to both wealth and health. Thus, a causal interpretation of these findings is not warranted. We did consider some possible nonshared confounders in adulthood that were related to lifestyle and disease, however. Sensitivity analyses restricted to participants in good midlife health suggested that these variables had a modest role in the wealth-longevity association.

Second, at MIDUS 1, a single self-reported questionnaire item was used to measure net worth, which may have introduced error into estimates of net worth. In addition, most participants in the MIDUS 1 sample self-identified as White individuals; thus, the estimates we reported may be less generalizable to underrepresented racial/ethnic groups. This limitation is particularly relevant for present purposes given the substantial and persistent racial disparities in household wealth in the US.^39^

Third, although we observed evidence of within-family associations between wealth and longevity, the mechanisms involved were not identified. Wealth is likely to be a distal factor in longevity, acting through more proximal mechanisms (eg, stress and lifestyle) to affect biological processes involved in disease as well as access to medical care and other health-promoting resources. This interpretation is consistent with the way other studies have conceptualized socioeconomic status as a fundamental cause of health disparities.^40,41^ From this perspective, having wealth allows individuals numerous opportunities to invest resources into the many proximal factors that promote health and longevity.

## Conclusions

This cohort study found an association between wealth at midlife and longevity, and this association is unlikely to be merely an artifact of environmental and heritable characteristics shared by families. Policies that support individuals' ability to accrue wealth and achieve financial security could have considerable health benefits. In addition, policies to reduce the wealth gap may generate substantial returns to public health.

## Notes

Supplement. **eMethods 1.** Full Methods Section **eMethods 2.** Supplementary Sensitivity Analyses **eMethods 3.** Deviations From Original Preregistered Analysis Plan **eTable 1.** Spline Model **eTable 2.** BW Model Among Siblings and Twins in Groups/Pairs Where All Family Members Have ≤$382,500 in Net Worth at M1 **eTable 3.** Multi-Level Cox Regression Analysis in Full Analysis Sample **eTable 4.** Multi-Level Cox Regression Analysis in Sample of Siblings and Twins **eTable 5.** Multi-Level Cox Regression Analysis Across Sibling Subsamples
eReferences.
