# Supplementary material for: Association of Wealth With Longevity in US Adults at Midlife
Source: JAMA Health Forum. 2021 Jul 23;2(7):e211652. doi: 10.1001/jamahealthforum.2021.1652 (PMC8796893; doi:10.1001/jamahealthforum.2021.1652)
Supplement: Supplement. — eMethods 1. Full Methods Section eMethods 2. Supplementary Sensitivity Analyses eMethods 3. Deviations From Original Preregistered Analysis Plan eTable 1. Spline Model eTable 2. BW Model Among Siblings and Twins in Groups/Pairs Where All Family Members Have ≤$382,500 in Net Worth at M1 eTable 3. Multi-Level Cox Regression Analysis in Full Analysis Sample eTable 4. Multi-Level Cox Regression Analysis in Sample of Siblings and Twins eTable 5. Multi-Level Cox Regression Analysis Across Sibling Subsamples eReferences. [file jamahealthforum-e211652-s001.pdf]

Supplementary Online Content

Finegood ED, Briley DA, Turiano NA, et al. Association of wealth with longevity in US adults at midlife. *JAMA Health Forum*. 2021;2(7):e211652. doi:10.1001/jamahealthforum.2021.1652

eMethods 1. Full Methods Section

eMethods 2. Supplementary Sensitivity Analyses

eMethods 3. Deviations From Original Preregistered Analysis Plan

eTable 1. Spline Model

eTable 2. BW Model Among Siblings and Twins in Groups/Pairs Where All Family

Members Have <= $382,500 in Net Worth at M1

eTable 3. Multi-Level Cox Regression Analysis in Full Analysis Sample

eTable 4. Multi-Level Cox Regression Analysis in Sample of Siblings and Twins

eTable 5. Multi-Level Cox Regression Analysis Across Sibling Subsamples

eReferences.

This supplementary material has been provided by the authors to give readers additional information about their work.

© 2021 Finegood ED et al. *JAMA Health Forum.*

eMethods 1. Full Methods Section

This report adheres to the STROBE guidelines for cohort studies[1]. The analyses presented here were conducted in 2020-2021 and preregistered on the Open Science Framework (https://osf.io/zyedp) on November 15, 2019. The pre-registration includes hypotheses, decision rules for inclusion, rationale for covariates, and statistical plan. Deviations are detailed in eMethods 3 of this Supplement.

Participants

Data come from the Midlife in the United States (MIDUS)[2] study, an ongoing national study of health and aging processes begun in 1994. In the current analysis, we utilized data from wave 1 of MIDUS (MIDUS I) collected from years 1994-1996. 7,108 adults aged 20-75 years participated at MIDUS I. They were recruited via a nationally representative random digit dialing sampling strategy, which included recruiting subsamples of siblings and twins. Those who provided oral informed consent to participate completed a telephone assisted survey as well as a mailed self-administered questionnaire. The Institutional Review Boards at the University of Wisconsin and Harvard Medical School approved the MIDUS study procedures. Mortality data through October 2018 were collected by the MIDUS study team at the University of Wisconsin.

Analysis sample

Of the 7,108 cases participating at MIDUS 1, n = 3,715 were designated for purposes of the current analysis as 'singletons', meaning that they did not have a sibling or a co-twin in MIDUS. 1,479 cases were non-twin full siblings, meaning that they had at least one non-twin full sibling in MIDUS. 1,914 were twins. Of these, n = 30 were missing zygosity information and

© 2021 Finegood ED et al. *JAMA Health Forum.*

were excluded from our analysis sample. Additionally, n = 61 did not have a co-twin in MIDUS so were designated as singletons in our analysis. In families that had both a pair of MZ twins and a pair of DZ twins, only the MZ pair was retained in our analysis sample (19 DZ twins excluded). We chose to retain the MZ pair as opposed to the DZ pair because the number of MZ pairs was lower overall than the number of DZ pairs. In families that had either two pairs of MZ twins or two pairs of DZ twins, one twin pair was retained at random (42 cases excluded from analysis sample). Given all of these criteria, n = 7,017 cases were considered for inclusion in our analysis sample (3,776 singletons; 1,479 full siblings; 1,064 DZ twins; and 698 MZ twins). Survival analyses were conducted among those individuals with complete data on mortality status, net worth, and the covariates (n = 5,414 cases: 2,675 singletons, 1,282 non-twin siblings, 864 DZ twins, 593 MZ twins). Twin or siblings who had complete data and who were in a pair with a co-twin/sibling that was missing data were included in this analytic sample of 5,414 although they were excluded from subsequent discordant twin/sibling analyses, resulting in 1,214 non-twin siblings, 740 DZs, and 536 MZs in the discordant twin/sibling analyses.

Missing data. Survival analyses were conducted among those individuals with complete data on mortality status, net worth, and the covariates. The amount of missing data among the analysis variables collected in the phone interview was low (0% to 4% missing). Of the 7,017 cases, n = 6,240 (88.9%) had completed both the phone interview and the self-administered questionnaire. Accordingly, missingness on the analysis variables collected in the self-administered questionnaire was higher (12% out of n = 7,017 were missing race/ethnicity data and 20.2% out of n = 7,017 were missing net worth data). In addition, 6 participants did not have mortality data as of October 31, 2018. Considering the missing data on all analysis

© 2021 Finegood ED et al. *JAMA Health Forum.*

variables, 5,414 cases had complete data (2,675 singletons, 1,282 non-twin siblings, 864 DZ twins, 593 MZ twins). Compared to those included in the analysis sample (n = 5,414), those who were excluded from the analysis sample (n = 1,603) were more likely to be non-white (χ^2(1, n = 6171) = 28.96, p < .001), to have had a previous heart condition (χ^2(1, n = 6997) = 4.62, p = .03), were older in age (t(2443.35) = -2.01, p = .04, and reported lower levels of parent education (t(2206.91) = 5.60, p < .001). There were no associations between inclusion status and sex, previous cancer diagnosis, smoking status, and regular alcohol consumption. Because our primary research question concerned *within*-sibling/twin associations between net worth and mortality risk, these sociodemographic and health-related factors related to inclusion status were considered less problematic.

Measures

Net worth. At MIDUS 1, participants responded to the following prompt: "Suppose you (and your spouse or partner) cashed in all your checking and savings accounts, stocks and bonds, real estate, sold your home, your vehicles, and all your valuable possessions. Then suppose you put that money toward paying off your mortgage and all your other loans, debts, and credit cards. Would you have any money left over after paying your debts or would you still owe money?" Participants reported how much that amount would be using binned response categories that specified ranges of dollars. Those who would still owe money were truncated at a value of $0. Individuals whose net worth exceeded $1,000,000 were truncated at that value. This truncation prior to MIDUS 1 data release was due to privacy and human subjects concerns. However, the MIDUS study does provide the percentage that had negative net worth, and this was 13%. On the other end, those with net worth over $1 million comprised only 2% of the

© 2021 Finegood ED et al. *JAMA Health Forum.*

sample. These bottom- and top-truncations, like any truncation, reduce the size of correlation and regression coefficients thereby creating an underestimate. Such a conservative bias was deemed acceptable.

Covariates. Survival models included several covariates selected a priori because of their known associations with wealth and health. These included participant age, self-reported race/ethnicity (analyzed here as non-white vs. white), sex (female vs. male), and self-reported history of cancer or heart disease, as diagnosed by a medical doctor. We also controlled for ever having smoked regularly or consumed alcohol regularly. Highest level of parents' education (an indicator of childhood socioeconomic status), was also included as a covariate. In the case that siblings/twins differed in their report of the highest level of parents' education, the higher of the discrepant responses was used for all siblings/twins within the same family.

All-cause mortality. Mortality follow-up was completed by the MIDUS study team at the University of Wisconsin, Madison. Date of death was obtained from various sources, including responses from relatives, other informant reports, newspaper or online obituaries, and the National Death Index (using the 15th day of the month, rather than the exact day of death, to maintain participant confidentiality). Survival time was the number of years between the date when the MIDUS 1 self-administered questionnaires were returned to the MIDUS study team (years 1995-96) and the date of death (otherwise, if alive, the censor date of Oct 31, 2018, which was the date of the MIDUS study team's latest mortality update).

Analysis plan

A series of Cox survival analyses were estimated using Stata 16[3]. To test the individual-level association between wealth and longevity, we first conducted analyses among all cases

© 2021 Finegood ED et al. *JAMA Health Forum.*

that had complete data on analysis variables utilizing robust SEs to account for dependence among family members (Model 1).

Next, we estimated survival models only within the sub-sample of twins and non-twin siblings. In Model 2, siblings and twins were treated "as individuals" but a shared frailty was included to model dependence among family members. In Model 3, we estimated the within-family association between wealth and longevity by calculating the family-level average net worth (among families that had >=2 members with complete data) and subsequently calculating the difference between each individual's net worth and their family average. When included alongside the family-level mean, the hazard ratio (HR) for these mean-deviation scores estimate the within-family association between net worth and longevity. This "between-within" (BW) method is a common approach to fixed-effects modeling[4,5]. When applied in this way, it allows us to compare siblings/twins in the same family to one another, and thus to control for all unmeasured shared family-level variables, consistent with the discordant sibling/twin design[6–8]. In survival analysis, the BW method has been shown to provide similar estimates to more common approaches for co-twin/sibling control with survival data (e.g. conditional likelihood methods like stratified Cox regression) and has been observed to be optimal statistically[5].

To further disambiguate environmental versus genetic influences, we tested whether within-family associations between net worth and longevity varied across non-twin sibling, MZ, and DZ subsamples. We did this by including a pair of two-way interaction terms crossing the mean-deviation scores of net worth with dummy codes for DZs or MZs; non-twin full siblings modeled as reference group). A Wald test tested the equality of the within-family net worth

© 2021 Finegood ED et al. *JAMA Health Forum.*

coefficient across siblings, DZ, and MZ subsamples. Lastly, we estimated separate survival models for non-twin siblings, DZ pairs, and MZ pairs (Models 4-6). A significant within-family association ($p < 0.05$) observed among non-twin siblings - but not among DZ or MZ pairs - would suggest some residual confounding by early life factors since twins share a closer pre- and post-natal environment than non-twin siblings who may be born years apart. A within-family association observed both among siblings and DZ pairs but not among MZ pairs would suggest genetic confounding. Sensitivity analyses were undertaken to test the robustness of findings. These analyses addressed the skewed distribution of the net worth variable and tested the possibility of non-linear associations between net worth and longevity. They also clarified the role of pre-existing health problems and considered other model specifications. Primary models were also re-estimated as stratified Cox regressions using STATA[3] (see eMethods 2) and as multilevel Cox regressions using Mplus[9] (see eMethods 3).

© 2021 Finegood ED et al. *JAMA Health Forum.*

eMethods 2. Supplementary Sensitivity Analyses

Sensitivity analyses were undertaken in the combined sibling and twin subsample to test the robustness of the findings to different model specifications. First, to assess the potential for a non-linear association between net worth and longevity, we estimated a spline model (eTable 1) including two knots: one at the 75th percentile of net worth ($125,000) and one at the 90th percentile ($382,500). As shown in eTable 1, HRs for net worth for those below the 75th percentile (HR=.86, CI=.76-.97, p=.01) and between the 75th-90th percentiles (HR=.90, CI=.82-.98, p=.01) were similar, and a subsequent test of the estimates confirmed that their difference was no greater than chance (p=.66). The HR for net worth for those above the 90th percentile (individuals with >=$680,000; n=182, 45 decedents) was not statistically significant (HR=1.02, 95%CI=.97-1.08, p=.31) and a test of the estimates indicated that the difference between this HR and the HR for those between the 75th-90th percentiles was statistically significant (p=.03). This indicates a possible diminished return on net worth at the very high end of the net worth distribution, though this reflects only approximately 7% of the sample. Collectively, the spline model indicates that among the large majority (93%) of siblings and twins (i.e. those whose net worth was <= $382,000), the association between net worth and survival was approximately linear.

We subsequently reran Model 3 in a subsample of siblings and twins who were in family groups where all members had <= $382,500 in net worth (n=2,110; 321 deaths). As shown in eTable 2 of this supplement, the HR for net worth in this restricted sample (HR=0.89, CI=0.82-0.96, p=0.004) suggested a larger association between net worth and longevity among those

© 2021 Finegood ED et al. *JAMA Health Forum.*

with lower family-level wealth. However, these results should be interpreted with a high level of caution due to the restricted sample size.

In another sensitivity analysis, we recoded net worth into ordinal decile groups, given the large positive skew of the net worth distribution. In the combined sibling and twin subsample, between-family (HR = 0.90, 95% CI = 0.85-0.94, $p < .001$) and within-family (HR = 0.92, 95% CI = 0.87-0.96, $p = .001$) net worth estimates remained significant predictors of mortality.

Next, to account for the possibility of residual confounding by health status (having a medical problem may both reduce one's ability to accumulate wealth and increase mortality risk), analyses were re-estimated among sibling/twin pairs who were free of previous cancer or heart disease. Among sibling groups with >2 members, only those siblings without heart disease and cancer were compared to one another. Results were largely similar in this restricted sample (n = 1,740; 196 deaths): $HR_{between}$ = 0.95, 95% CI= 0.90 - 0.99, $p = 0.04$, and HR_{within} = 0.94, 95% CI = 0.90-0.98, $p = 0.01$).

We also tested the possibility of a nonlinear age trend by including an age^2 term (HR =1.00, 95% CI = 0.99-1.00, $p = .65$) and also an age*sex interaction term (HR = 1.01, 95% CI = 0.99-1.03, $p = 0.14$), neither of which was associated with mortality risk nor changed the interpretations of other model estimates. We also tested an interaction between the within-family net worth estimate and participant age at MIDUS 1. The rationale being that the within-family association between net worth and longevity may vary as a function of age. The interaction term was not statistically significant, HR = 1.00, 95% CI =0.99-1.00, p=0.37,

© 2021 Finegood ED et al. *JAMA Health Forum.*

suggesting that the within-family association between net worth and longevity did not vary by age at MIDUS 1.

Lastly, as a more conservative test of possible confounding by early experience, we restricted the analysis sample to only same-sex sibling groups/twin pairs. Point estimates of between-family (HR = .94, 95% CI = 0.90-0.98 *p* = .007) and within-family (HR = .96, 95% CI = 0.92-1.01, *p* = .16) net worth estimates were consistent with estimates observed in Model 3 in the main text, although the p-value for the within-family estimate was not statistically significant, likely due to the substantial reduction in power and sample size in this restricted sample (N_{Model3}=2,490; n=421 dead; vs. $N_{Same\text{-}sex}$ = 1,359; n=221 dead). Given the large loss in power due to the reduction in sample size and number of deaths, the result of this sensitivity analysis should be interpreted with a high level of caution.

Stratified Cox Regression models. We re-ran Models 3-6 as stratified Cox regressions, stratifying by Family ID. The within-family net worth estimates were as follows: Model 3 HR = .94, 95%CI = .90-.97, p = .002; Model 4 HR = .93, 95%CI = .89-.97, p = .004; Model 5 HR = .95, 95%CI = .87-1.05, p = .37; Model 6 HR = .95, 95%CI = .84-1.07, p = .42.

© 2021 Finegood ED et al. *JAMA Health Forum.*

eMethods 3. Deviations From Original Preregistered Analysis Plan

The analyses presented in the main text are consistent with the study rationale, hypotheses, and overall analytic plan outlined in our preregistration (https://osf.io/zyedp). We did, however, deviate from the original analytic plan in some minor ways—these deviations are outlined below.

First, in the original analysis plan, we specified that survival analyses would be estimated as multi-level Cox regression models. Instead, each survival model was estimated as a Cox model with shared frailty term to account for clustering. Both analytic approaches gave very similar results for all models—compare coefficients in Model 1 and Model 3 of Table 2 in the main text to estimates in eTables 3 and 4 (results from multilevel Cox regressions run in Mplus version 8[9]).

Second, for the sibling/twin comparison analysis (step 2 of the original analysis plan), we originally planned to run a two-level mixture analysis using a Cox regression model specifying the KNOWNCLASS option in Mplus version 8 in order to estimate a multiple-group analysis—allowing model coefficients to vary across subsamples of non-twin siblings, DZ twins, MZ twins. Instead, in the main text, we present results from three separate survival models: one among non-twin siblings, one among DZ twins, and one among MZ twins. With either analytic strategy, coefficients were very similar and provided the same interpretations—compare coefficients from Models 4, 5, and 6 in main text Table 2 to coefficients in eTable 5, which display results from the multilevel mixture model using Cox regression with KNOWNCLASS option in Mplus version 8).

© 2021 Finegood ED et al. *JAMA Health Forum.*

Third, as described in the main text, we undertook a conservative test of whether the within-family net worth effect varied across full sibling, MZ twin, and DZ twin subsamples by including two-way interaction terms between the within-family net worth variable and dummy codes for cluster type and conducting a Wald test to test the equality of the within-family estimates. This test of the equality of within-family net worth effects across sibling subsamples was not described in the original analysis plan.

We also originally proposed an exploratory test of the two-way interaction between parent education and the within-family net worth twin/sibling difference score. We decided to omit this test from the main text because we felt that the rationale for this test was tangential to our primary analyses, which were already many in number. It is also not an optimal test for answering questions related to social mobility, its stated purpose in the preregistration. In any case, we report results from this test here. Among the combined sample of non-twin siblings, DZ twins, and MZ twins (n =2,490) there was no interaction between parents' highest level of education and sibling/twin net worth difference score (HR = .99, 95% CI .98- 1.00, *p* = .30). Indeed, when the sample is split into subsamples of those at or below (n = 1,333) and above (n = 1,157) the median on parent education, the within-family net worth estimate was significant in both subsamples (at/below median: HR_{within}=.95, 95% CI .91-.99, *p* = .04; above median: HR_{within}=.94, 95% CI .89-.98, *p* = .006).

We also specified that full-information maximum likelihood estimation (FIML) would be used to handle missing data. In the preregistration, we specified the criteria by which siblings and twin pairs would be excluded from our analysis sample—resulting in an analytic sample of n=7,017. As described in eMethods 1 of the Supplement, of the n=7,017, n=6,240 had

© 2021 Finegood ED et al. *JAMA Health Forum.*

completed both the phone interview and the self-administered questionnaire. Another n = 6 did not have mortality data, resulting in a possible analytic sample of n=6,234. Of the analysis variables, net worth had the most missingness: n=640 out of 6,234 (10.3%) missing net worth, likely because some participants were unwilling or unable to provide this information. Because our primary interest was to compare mortality risk within sibling groups/twin pairs who were discordant on net worth, only siblings groups/twin pairs in which discordance could be estimated (e.g. twin pairs in which both twins had non-missing net worth data) were useful analytically. Thus, analyses were conducted only among cases that had complete data and FIML was not used.

© 2021 Finegood ED et al. *JAMA Health Forum.*

eTable 1. Spline Model

	HR	95% CI	p
Age	1.11	1.09-1.12	< .001
Female	0.74	0.60-0.93	.009
Non-white	0.82	0.46-1.46	0.51
Parent education	0.97	0.93-1.01	0.18
Heart disease	1.96	1.56-2.47	< .001
Cancer	1.57	1.18-2.07	0.002
Smoking	2.07	1.65-2.60	< .001
Alcohol use	0.92	0.74-1.15	0.50
Net worth spline 1	0.86	0.76-0.97	0.01
Net worth spline 2	0.90	0.82-0.98	0.01
Net worth spline 3	1.02	0.97-1.08	0.31

Note: N=2,490, which includes 421 deaths. HR=hazard ratio, 95% CI= 95% confidence interval of the hazard ratio.

© 2021 Finegood ED et al. *JAMA Health Forum.*

eTable 2. BW Model Among Siblings and Twins in Groups/Pairs Where All Family Members Have <= $382,500 in Net Worth at M1

	HR	95% CI	p
Age	1.11	1.09-1.12	< 0.001
Female	0.74	0.58-0.95	0.01
Non-white	0.67	0.37-1.23	0.20
Parent education	0.97	0.92-1.02	0.27
Heart disease	2.04	1.58-2.63	< 0.001
Cancer	1.56	1.14-2.14	0.005
Smoking	1.87	1.45-2.41	< 0.001
Alcohol use	1.04	0.81-1.34	0.71
Net worth (between-family)	0.84	0.78-0.90	< 0.001
Net worth (within-family)	0.89	0.82-0.96	0.004

Note: N=2,110, which includes 321 deaths. HR=hazard ratio, 95% CI= 95% confidence interval of the hazard ratio.

eTable 3. Multi-Level Cox Regression Analysis in Full Analysis Sample

	HR (95% CI)	p-value
Age	1.10 (1.09-1.11)	< 0.001
Female	0.82 (0.72-0.94)	0.005
Non-white	1.13 (0.87-1.46)	0.35
Parent education	0.97 (0.95-0.99)	0.03
Heart disease	1.87 (1.61-2.17)	< 0.001
Cancer	1.44 (1.22-1.70)	< 0.001
Smoking	1.75 (1.53-2.00)	< 0.001
Alcohol use	1.04 (0.91-1.19)	0.47
Net worth	0.95 (0.94-0.97)	< 0.001

© 2021 Finegood ED et al. *JAMA Health Forum.*

eTable 4. Multi-Level Cox Regression Analysis in Sample of Siblings and Twins

	HR (95% CI)	p-value
Age	1.10 (1.09-1.11)	< 0.001
Female	0.78 (0.64-0.96)	0.01
Non-white	0.85 (0.48-1.50)	0.58
Parent education	0.97 (0.93-1.01)	0.25
Heart disease	1.85 (1.46-2.35)	< 0.001
Cancer	1.53 (1.18-1.99)	0.001
Smoking	2.03 (1.63-2.52)	< 0.001
Alcohol use	0.92 (0.74-1.14)	0.46
Net worth (between-family)	0.96 (0.93-0.99)	0.01
Net worth (within-family)	0.95 (0.92-0.98)	0.002

© 2021 Finegood ED et al. *JAMA Health Forum.*

eTable 5. Multi-Level Cox Regression Analysis Across Sibling Subsamples

	Siblings (n = 1,214)			DZ twins (n = 740)			MZ twins (n = 536)	
	HR (95% CI)	p-value		HR (95% CI)	p-value		HR (95% CI)	p-value
Age	1.10 (1.08-1.12)	< 0.001		1.12 (1.09-1.14)	< 0.001		1.11 (1.08-1.15)	< 0.001
Female	0.64 (0.47-0.87)	0.005		0.88 (0.60-1.29)	0.51		1.03 (0.58-1.83)	0.89
Non-white	1.50 (0.66-3.42)	0.32		0.25 (0.05-1.09)	0.06		1.16 (0.31-4.29)	0.81
Parent edu.	1.00 (0.94-1.06)	0.87		0.96 (0.90-1.03)	0.38		0.91 (0.82-1.02)	0.11
Heart disease	2.07 (1.51-2.86)	< 0.001		1.72 (1.09-2.69)	0.01		2.39 (1.10-5.21)	0.02
Cancer	1.92 (1.37-2.70)	< 0.001		1.01 (0.57-1.77)	0.96		1.55 (0.58-4.14)	0.38
Smoking	2.21 (1.63-2.98)	< 0.001		2.08 (1.39-3.11)	< 0.001		2.11 (1.17-3.80)	0.01
Alcohol use	0.73 (0.52-1.01)	0.06		1.18 (0.81-1.72)	0.36		1.04 (0.61-1.76)	0.86
Net worth (between)	0.98 (0.93-1.02)	0.41		0.90 (0.84-0.97)	0.005		0.95 (0.89-1.03)	0.26
Net worth (within)	0.94 (0.90-0.98)	0.005		0.94 (0.86-1.02)	0.19		0.95 (0.90-1.01)	0.16

© 2021 Finegood ED et al. *JAMA Health Forum.*

eReferences

1. von Elm E, Altman DG, Egger M, et al. The Strengthening the Reporting of Observational Studies in Epidemiology (STROBE) Statement: Guidelines for Reporting Observational Studies. *PLoS Med*. 2007;4(10):e296. doi:10.1371/journal.pmed.0040296

2. Brim OG, Ryff CD, Kessler RC, eds. *How Healthy Are We? A National Study of Well-Being at Midlife*. Chicago, IL: University of Chicago Press; 2004.

3. StataCorp. *Stata Statistical Software: Release 16*. StataCorp LLC; 2019.

4. Allison PD. *Fixed Effects Regression Models*. Sage Publications, Inc.; 2009.

5. Sjölander A, Lichtenstein P, Larsson H, Pawitan Y. Between-within models for survival analysis. *Stat Med*. 2013;32(18):3067-3076. doi:10.1002/sim.5767

6. Carlin JB, Gurrin LC, Sterne JAC, Morley R, Dwyer T. Regression models for twin studies: A critical review. *Int J Epidemiol*. 2005;34(5):1089-1099. doi:https://doi.org/10.1093/ije/dyi153

7. Turkheimer E, Harden KP. Behavior Genetic Research Methods: Testing Quasi-Causal Hypotheses Using Multivariate Twin Data. In: Reis HT, Judd CM, eds. *Handbook of Research Methods in Social and Personality Psychology*. 2nd ed. Cambridge University Press; 2014:159-187.

8. McGue M, Osler M, Christensen K. Causal inference and observational research: The utility of twins. *Perspect Psychol Sci*. 2010;5(5):546-556. doi:10.1177/1745691610383511

9. Muthén LK, Muthén BO. *Mplus User's Guide*. Eighth Ed. Los Angeles, CA: Muthén & Muthén. 1998-2017.

© 2021 Finegood ED et al. *JAMA Health Forum.*
